# Melatonin alleviates angiotensin-II-induced cardiac hypertrophy via activating MICU1 pathway

**DOI:** 10.18632/aging.202159

**Published:** 2020-11-26

**Authors:** Yi Yang, Jin Du, Rui Xu, Yang Shen, Dachun Yang, De Li, Houxiang Hu, Haifeng Pei, Yongjian Yang

**Affiliations:** 1 Department of Cardiology, The General Hospital of Western Theater Command, Chengdu 610083, China; 2 Department of Cardiology, Affiliated Hospital of North Sichuan Medical College, Nanchong 637000, China

**Keywords:** cardiac hypertrophy, melatonin, PGC-1α, MICU1, ROS

## Abstract

Mitochondrial calcium uptake 1 (MICU1) is a pivotal molecule in maintaining mitochondrial homeostasis under stress conditions. However, it is unclear whether MICU1 attenuates mitochondrial stress in angiotensin II (Ang-II)-induced cardiac hypertrophy or if it has a role in the function of melatonin. Here, small-interfering RNAs against MICU1 or adenovirus-based plasmids encoding MICU1 were delivered into left ventricles of mice or incubated with neonatal murine ventricular myocytes (NMVMs) for 48 h. MICU1 expression was depressed in hypertrophic myocardia and MICU1 knockdown aggravated Ang-II-induced cardiac hypertrophy *in vivo* and *in vitro*. In contrast, MICU1 upregulation decreased cardiomyocyte susceptibility to hypertrophic stress. Ang-II administration, particularly in NMVMs with MICU1 knockdown, led to significantly increased reactive oxygen species (ROS) overload, altered mitochondrial morphology, and suppressed mitochondrial function, all of which were reversed by MICU1 supplementation. Moreover, peroxisome proliferator-activated receptor gamma coactivator 1-α (PGC-1α)/MICU1 expression in hypertrophic myocardia increased with melatonin. Melatonin ameliorated excessive ROS generation, promoted mitochondrial function, and attenuated cardiac hypertrophy in control but not MICU1 knockdown NMVMs or mice. Collectively, our results demonstrate that MICU1 attenuates Ang-II-induced cardiac hypertrophy by inhibiting mitochondria-derived oxidative stress. MICU1 activation may be the mechanism underlying melatonin-induced protection against myocardial hypertrophy.

## INTRODUCTION

As an independent risk factor for cardiac morbidity and mortality, cardiac hypertrophy represents a pathological adaptation for pressure overload and hypertension [1, 2]. In hearts, mitochondria are central to cardiac stress responses [3]. Convincing evidence reveals that obvious mitochondrial abnormalities, such as pronounced changes in the composition and function of mitochondria proteome, were observed in pathological cardiac hypertrophy [4, 5]. However, the molecular mechanism of the mitochondrial alterations underlying cardiac hypertrophy has not been clearly defined yet.

Calcium entry into the mitochondria is critical in maintaining cellular homeostasis [6] and is believed to modulate bioenergetic capacity and help determine the threshold for cell death [7, 8]. It is well known that mitochondrial calcium channels are macromolecular complexes, basically consisting of the pore-forming mitochondrial calcium uniporter (MCU) protein, the essential MCU regulator, and the mitochondrial calcium uptake 1 (MICU1) [9]. MICU1 is a Ca^2+^ binding protein that resides in the mitochondrial intermembrane space and suggested to be required for uniporter-mediated Ca^2+^ uptake [10]. In particular, it has been reported that a loss-of-function mutations in MICU1 cause a brain and muscle disorder [11]. Xue et al. [12] showed that MICU1 protects against myocardial ischemic/reperfusion (MI/R) injury and is controlled by the importer receptor Tom70 in the mitochondrial outer membrane complex. Moreover, Ji et al. [13] have demonstrated that MICU1 exhibits protective effects in diabetic cardiomyopathy. To our knowledge, the role of MICU1 in angiotensin II (Ang-II)-induced cardiac hypertrophy, has never been reported.

Oxidative stress occurs when excess reactive oxygen species (ROS) are generated that cannot be adequately countered by intrinsic antioxidant systems [14]. Excessive ROS generation triggers cell dysfunction, lipid peroxidation, and DNA mutagenesis and can lead to cell damage or death [15]. ROS has been identified as one of the key contributing factors in the development of cardiac hypertrophy [16]. Cumulative evidence suggests that ROS-sensitive signaling regulated by stress inducers such as Ang-II, endothelin-1 and tumor necrosis factor alpha have a causative and prominent role in the cardiac hypertrophy [17]. Interestingly, recent studies have found that MICU1 deficiency renders mitochondria to constitutively load with Ca^2+^, leading to excessive generation of ROS and high sensitivity to apoptotic stress [6]. Furthermore, it has been reported that the Akt-mediated phosphorylation impairs MICU1 processing and stability, culminating in ROS production and tumor progression [18]. However, whether MICU1 exerts antioxidant effects on hypertrophic hearts is still unknown.

As an endogenous hormone produced by the pineal gland, melatonin, chemically N-acetyl-5-methoxytryptamine, is released exclusively at night [19, 20]. Epidemiological studies revealed that melatonin secretion in pineal, as well as circulating melatonin level, is reduced in heart failure (HF) patients [21]. Meanwhile melatonin levels were also associated with reverse remodeling after cardiac resynchronization therapy in patients with HF [22]. Melatonin is a ubiquitous and versatile molecule that exhibits most of the desirable characteristics of good antioxidant. The amount of data gathered so far regarding the protective action of melatonin against oxidative stress is overwhelming [23]. Therefore, melatonin may exert protective effects by decreasing ROS overload. However, whether melatonin can influence MICU1 expression in hypertrophic myocardium remains unclear.

Therefore, the present study was designed to identify the following: (i) whether MICU1 expression is altered in Ang-II-induced cardiac hypertrophy, (ii) whether MICU1 deficiency aggravates cardiac hypertrophy, and if so, (iii) whether melatonin suppresses oxidative stress and attenuates mitochondrial abnormalities in cardiac hypertrophy by activating MICU1.

## RESULTS

### Mitochondrial Ca^2+^-sensing regulators were downregulated in hypertrophic hearts

**Figure 1:**
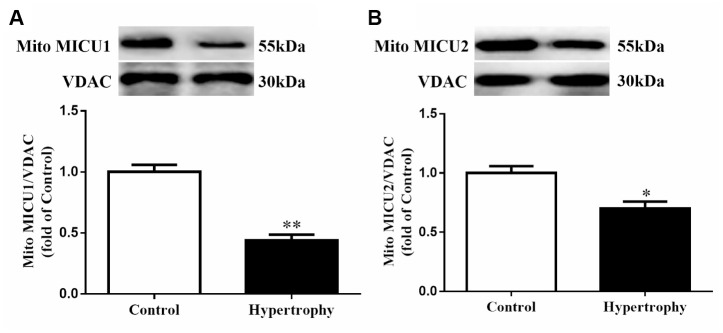
**The expressions of mitochondrial MICUs were reduced in Ang-II-induced hypertrophic hearts.** (**A**, **B**) Western blotting was used to determine the protein expression levels of mitochondrial MICU1 (**A**) and MICU2 (**B**) in control and hypertrophic mouse hearts. MICUs, mitochondrial calcium uniporter; MICU1, mitochondrial calcium uptake 1; MICU2, mitochondrial calcium uptake 2. VDAC, voltage-dependent anion channel. VDAC was used as a loading control. Presented values are means ± SEM. N=6-8/group. ^*^P<0.05, ^**^P<0.01 vs. Control.

To investigate the alterations of Ca^2+^-sensing regulators (MICUs) in hypertrophic hearts, the expression of mitochondrial MICU1 and its paralog MICU2 were determined. Western blotting demonstrated that MICU1 expression was significantly reduced in hypertrophic myocardium (Figure 1A). Meanwhile, we also measured mitochondrial MICU2 expression in cardiac hypertrophy. As a result, we found that the protein levels of mitochondrial MICU2 were also depressed in hypertrophic hearts, but its reduction was much less than MICU1 (Figure 1B). Collectively, these results indicated that MICUs, especially mitochondrial MICU1, might take part in the Ang-II-induced cardiac hypertrophy.

### MICU1 deficiency in the heart blunted Ang-II-induced cardiac hypertrophy

**Figure 2:**
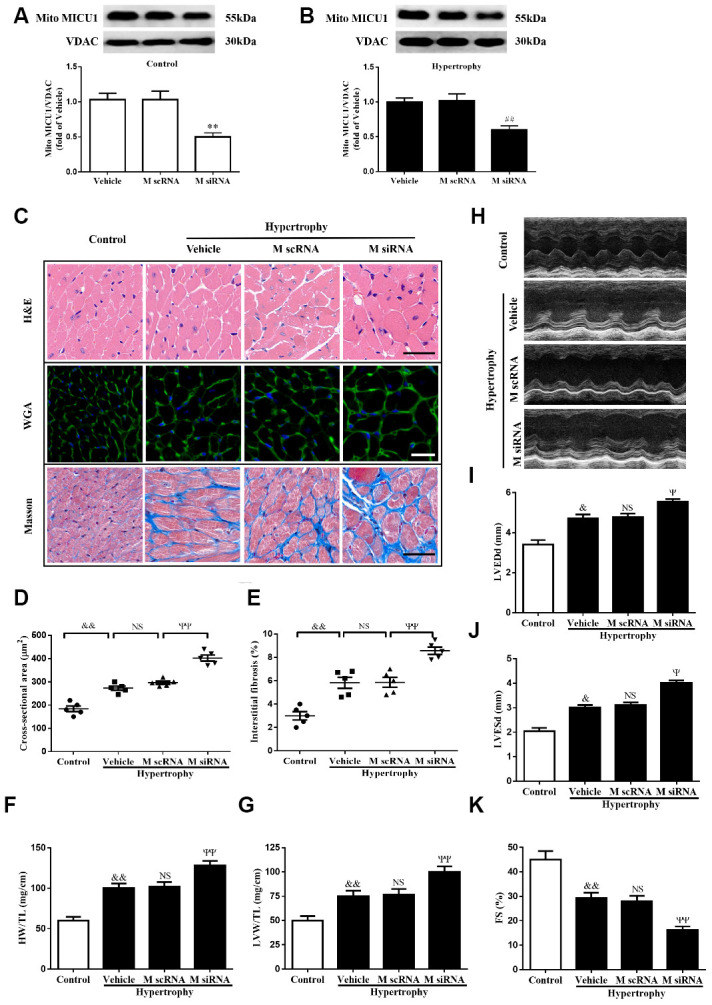
**MICU1 downregulation in the heart aggravated Ang-II-induced cardiac hypertrophy.** (**A**) Western blotting was used to measure the transfection efficiency of MICU1 siRNA in control mice. (**B**) Transfection efficiency of MICU1 siRNA in mice subjected to Ang-II was determined by Western blotting. (**C**) The heart sections stained with hematoxylin-eosin (H&E, the first row; Scale bars=50 μm), wheat germ agglutinin (WGA, the second row; Scale bars=20 μm) and Masson (the third row; Scale bars=50 μm) from the indicated groups were measured. (**D**) Cross-sectional cardiomyocyte areas were summarized. (**E**) The interstitial fibrosis was quantified. (**F**, **G**) The ratio of heart weight to tibia length (HW/TL) (**F**) and left ventricular weight to tibia length (LVW/TL) (**G**) were determined in different mice. (**H**) Representative echocardiographic image of the left ventricle in different mice was represented. (**I**–**K**) Echocardiographic assessment of left ventricular end-diastolic dimension (LVEDd) (**I**), left ventricular end-systolic dimension (LVESd) (**J**) and fractional shortening (FS) (**K**) was used to reflect cardiac function. M scRNA, scrambled siRNA; M siRNA, MICU1-specific siRNA. All the data represent the means ± SEM. N=6-8/group. ^**^P<0.01 vs. Vehicle in Control; ^##^*P*<0.01 vs. Vehicle in Hypertrophy; ^&^*P*<0.05, ^&&^*P*<0.01 vs. Control; ^Ψ^*P*<0.05, ^ΨΨ^*P*<0.01 vs. M scRNA of hypertrophy.

To examine concrete role of MICU1 in Ang-II-induced cardiac hypertrophy, we knocked down MICU1 in WT mice and hypertrophic mice by intramyocardial injection of MICU1-targeted small interfering RNA (siRNA) (Figure 1A, 1B). The results showed that the systolic blood pressure (SBP) and diastolic blood pressure (DBP) were significantly increased in both mouse models after Ang-II infusion. Meanwhile, blood pressure was indistinguishable between MICU1 knockdown and WT mice (Supplementary Figure 1A, 1B), suggesting that MICU1 do not affect Ang-II-induced hypertension. Moreover, MICU1 downregulation did not induce obviously hypertrophic injury (Supplementary Figure 1C, 1E) and we did not find any changes in basal physiological parameters in MICU1 knockdown mice (Supplementary Table 1). Histological analysis with hematoxylin-eosin (H&E), wheat germ agglutinin (WGA) or Masson staining revealed that the cardiomyocyte hypertrophy (Figure 2C, 2D) and interstitial fibrosis induced by Ang-II was markedly aggravated in MICU1 knockdown mice compared with the corresponding controls (Figure 2C, 2E). MICU1 downregulation showed significantly increased ratios of heart weight (HW)/tibia length (TL) and left ventricular weight (LVW)/TL (Figure 2F–2G). Furthermore, MICU1 reduction remarkably exacerbated cardiac dilation and dysfunction on the echocardiography assay, as evidenced by increased left ventricular end-diastolic dimension (LVEDd) and left ventricular end-systolic dimension (LVESd) and decreased fractional shortening (FS) (Figure 2H–2K). These data demonstrated that MICU1 ablation in the heart aggravated Ang-II-induced cardiac hypertrophy.

### Reduction in MICU1 aggravated Ang-II-induced cardiomyocyte hypertrophy *in vitro*

**Figure 3:**
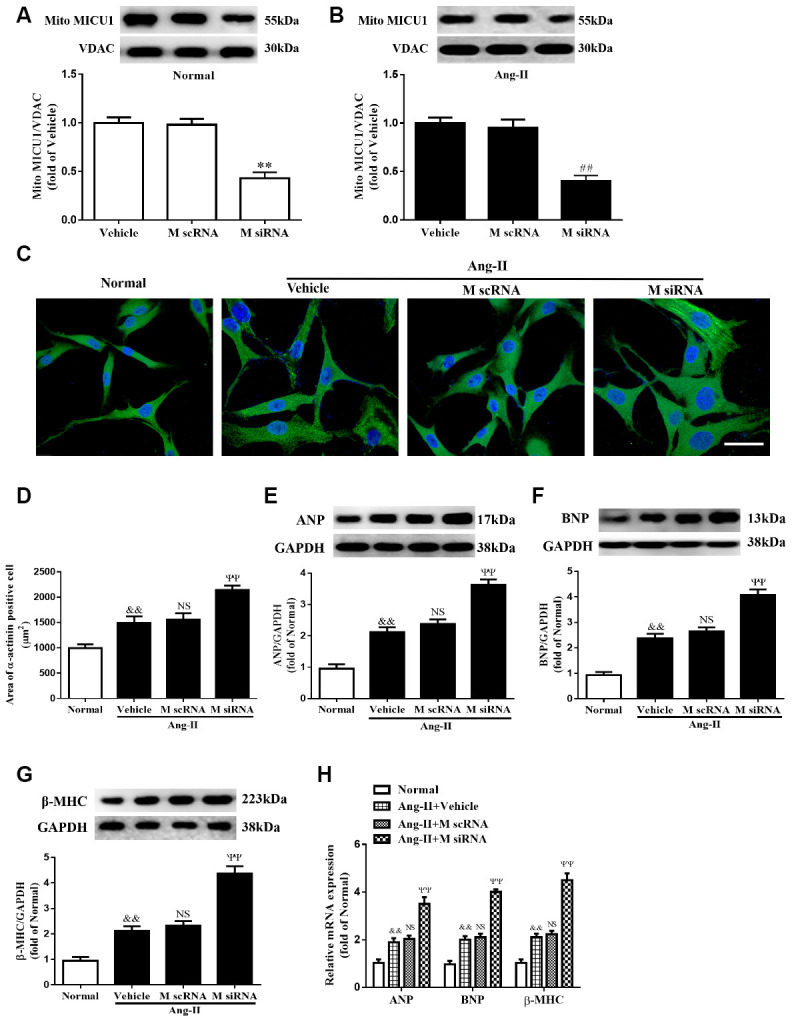
**Knockdown of MICU1 exacerbated Ang-II-induced cardiomyocyte hypertrophy *in vitro*.** (**A**, **B**) Western blotting was used to measure the transfection efficiency of MICU1 siRNA in neonatal mice ventricular myocytes (NMVMs) (**A**) and Ang-II treated NMVMs (**B**). (**C**, **D**) Cell surface areas were measured in neonatal mice ventricular myocytes (NMVMs) stimulated with Ang-II (**C**) and representative images of α-actinin (red)-and DAPI (blue)-stained cardiomyocytes were followed by cell area quantifications (**D**). Scale bars=10 μm. (**E**–**G**) Western blotting was used to measure protein levels of ANP (**E**), BNP (**F**) and β-MHC (**G**) in NMVMs. (**H**) The mRNA expression of ANP, BNP and β-MHC in NMVMs was detected by qRT-PCR. ANP, atrial natriuretic peptide; BNP, brain natriuretic peptide; β-MHC, β-myosin heavy chain. All the data represent the means ± SEM. N=6-8/group. ^**^P<0.01 vs. Vehicle in Normal; ^##^*P*<0.01 vs. Vehicle in Ang-II; ^&&^*P*<0.01 vs. Normal; ^ΨΨ^*P*<0.01 vs. M scRNA of Ang-II.

Considering that mice models of cardiac hypertrophy were complex, neonatal murine ventricular myocytes (NMVMs) were isolated from left ventricular in mice hearts (Supplementary Figure 2A), infected with specific MICU1 siRNA (Figure 3A, 3B), and used the well-established NMVMs models of hypertrophy to define the specific role of MICU1 in cardiomyocytes. The decrease in MICU1 levels by siRNA led to a clear increase in cell surface area (Figure 3C, 3D). Furthermore, *in vitro* studies revealed that knockdown of MICU1 also elevated protein (Figure 3E, 3F) and mRNA (Figure 3G) levels of atrial natriuretic peptide (ANP), brain natriuretic peptide (BNP) and β-myosin heavy chain (β-MHC) in cardiomyocytes. These observations showed that knockdown of MICU1 exacerbated cardiomyocyte hypertrophy.

### Cardiac MICU1 overexpression alleviated cardiac hypertrophy

**Figure 4:**
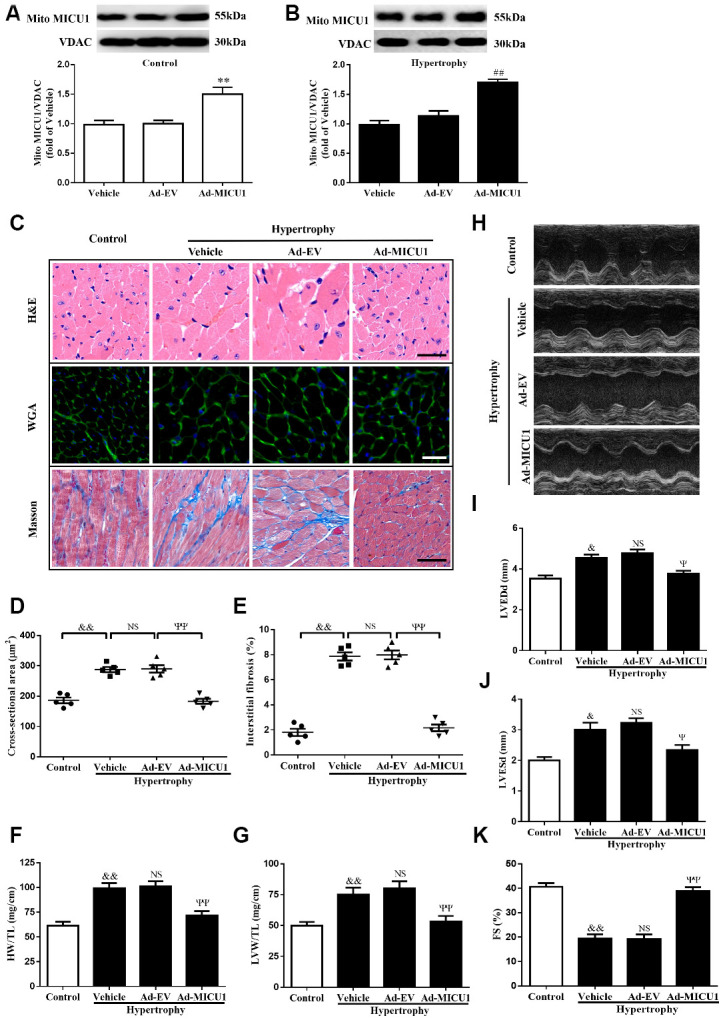
**MICU1 overexpression in the heart attenuated cardiac hypertrophy.** (**A**, **B**) Representative immunoblots and quantification of protein levels of MICU1 in control (**A**) and hypertrophy hearts (**B**) infected with Ad-EV and Ad-MICU1 were shown. (**C**) Histological analyses of heart sections stained with H&E, wheat germ agglutinin, and Masson from the indicated groups were presented. (**D**) The average cross-sectional area of cardiomyocytes from the indicated groups was summarized. (**E**) The interstitial fibrosis was quantified. (**F**, **G**) The rations of HW/TL (**F**) and LVW/TL (**G**) in different mice were determined. (**H**) Representative echocardiographic image of the left ventricle in different mice was represented. (**I**–**K**) Echocardiographic assessment of LVEDd, LVESd and FS was used to reflect cardiac function. Ad-EV, control adenovirus; Ad-MICU1, recombinant adenovirus encoding MICU1. All the data represent the means ± SEM. N=6-8/group. ^**^P<0.01 vs. Vehicle in Control; ^##^*P*<0.01 vs. Vehicle in Hypertrophy; ^&^*P*<0.05, ^&&^*P*<0.01 vs. Control; ^Ψ^*P*<0.05, ^ΨΨ^*P*<0.01 vs. Ad-EV of hypertrophy.

To confirm the causative effects of MICU1 in cardiac hypertrophy, an adenoviral vector expressing MICU1 was administered through direct injection into left ventricle in WT and hypertrophic mice. Following adenoviral injection, MICU1 expression in ventricular myocardium was confirmed by using Western blotting analysis (Figure 4A, 4B). Intramyocardial injection of an adenoviral vector expressing MICU1 did not induce adverse effects in mice (Supplementary Figure 3A–3D) and basal physiological parameter (Supplementary Table 1) and related indexes of hypertrophy (Supplementary Figure 3E–3G) were indistinguishable in between MICU1 overexpression and WT mice. Notably, MICU1 supplementation ameliorated the enlargement of cardiomyocytes, attenuated cardiac interstitial fibrosis (Figure 4C–4E), decreased the ratios of HW/TL and LVW/TL (Figure 4F–4G), and rescued cardiac dilation function (Figure 4H–4K) in hypertrophic myocardium. Taken together, these findings revealed that MICU1 was sufficient to blunt Ang-II-induced cardiac hypertrophy *in vivo*.

### Overexpression of MICU1 ameliorated cardiomyocyte hypertrophy *in vitro*

**Figure 5:**
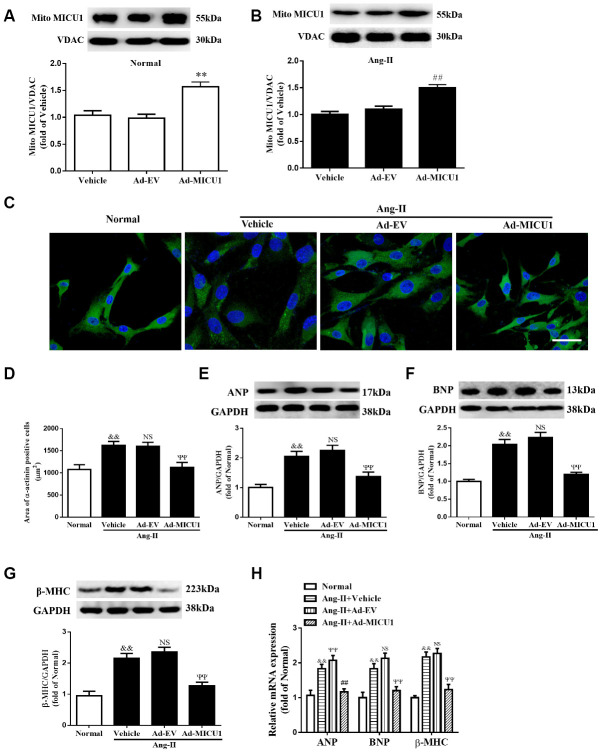
**Adenoviral overexpression of MICU1 was resistant to cardiomyocyte hypertrophy *in vitro*.** (**A**, **B**) Representative immunoblots and quantification of protein levels of MICU1 in (NMVMs) (**A**) and Ang-II treated NMVMs (**B**) infected with Ad-EV and Ad-MICU1 were shown. (**C**, **D**) Cell surface areas were measured in NMVMs stimulated with Ang-II (**C**) and representative images of α-actinin (red)-and DAPI (blue)-stained cardiomyocytes (left) were followed by cell area quantifications (**D**). Scale bars=10 μm. (**E**–**H**) Western blotting and qRT-PCR were used to measure protein levels of ANP, BNP and β-MHC in NMVMs and Ang-II treated NMVMs. All the data represent the means ± SEM. N=6-8/group. ^**^P<0.01 vs. Vehicle in Normal; ^##^*P*<0.01 vs. Vehicle in Ang-II; ^&&^*P*<0.01 vs. Normal; ^ΨΨ^*P*<0.01 vs. Ad-EV of Ang-II.

Next, we evaluated whether cardiomyocyte MICU1 provide protection against the pathological hypertrophic growth induced by Ang-II. We found that the adenovirus-mediated overexpression of MICU1 (Figure 5A, 5B) ablated the hypertrophic responses of NMVMs to Ang-II, as evidenced by the restoration of cell surface area (Figure 5C, 5D), decreased expression of protein and mRNA levels of ANP, BNP and β-MHC (Figure 5E–5H). Together, these data showed that MICU1 indeed alleviated cardiomyocyte hypertrophy *in vitro*.

### Oxidative stress mediated MICU1 deficiency-induced cardiomyocyte hypertrophy

**Figure 6:**
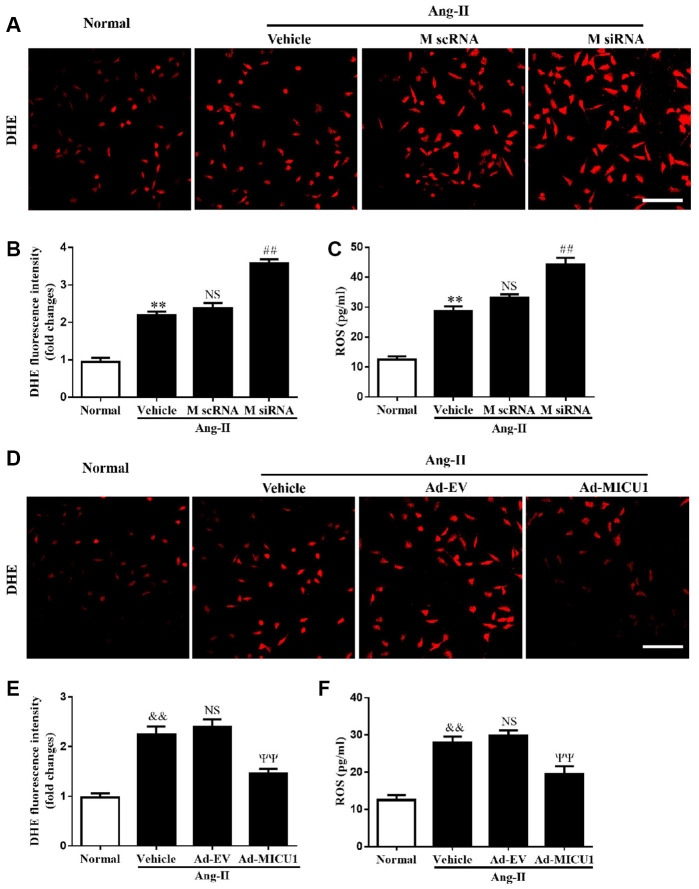
**MICU1 mediated cardiomyocyte hypertrophy by modulating oxidation states.** (**A**, **B**) The ROS levels in cardiomyocytes treated with Ang-II and MICU1 siRNA were analyzed by DHE staining. Representative confocal microscope images (**A**) and fluorescence quantitation (**B**) were presented. Scale bars=10 μm. (**C**) ROS generation in cardiomyocytes treated with Ang-II and MICU1 siRNA was detected by an ELISA kit. (**D**, **E**) The ROS levels in NMVMs treated with Ang-II and Ad-MICU1 were analyzed by DHE staining. Representative confocal microscope images (**D**) and fluorescence quantitation (**E**) were presented. Scale bars=10 μm. (**F**) ROS generation in cardiomyocytes treated with Ang-II and Ad-MICU1 was detected by an ELISA kit. Presented values are means ± SEM. N=6-8/group. ^**^P<0.01 vs. Normal (1); ^##^*P*<0.01 vs. M scRNA of Ang-II; ^&&^*P*<0.01 vs. Normal (2); ^ΨΨ^*P*<0.01 vs. Ad-EV of Ang-II.

Oxidative stress is a common mechanism underlying the pathological cardiac hypertrophy, and MICU1 might exert an antioxidant effect on hypertrophic growth stimulated by Ang-II. To identify whether MICU1 has an antioxidant role in hypertrophic NMVMs, ROS level was determined by the average DHE staining intensity, ELISA kits and fluorescent probe MitoSOX. As shown in Figure 6A–6C, Ang-II induced an overt elevation of ROS generation in NMVMs, which was further aggravated by MICU1 reduction. To confirm the injury-causing roles of oxidative stress and MICU1 deficiency, we investigated effects of MICU1 overexpression on ROS in NMVMs. As a result, MICU1 overexpression remarkedly decreased ROS generation (Figure 6D–6F). A similar pattern of mitochondrial ROS (MitoROS) level was found in Ang-II-induced cardiomyocyte hypertrophy (Supplementary Figure 4A–4D). Thus, the *in vitro* data indicated that oxidative stress was a hypertrophic mediator of the effects of MICU1 deficiency.

### Knockdown of MICU1 aggravated cardiac hypertrophy-induced mitochondrial injury

**Figure 7:**
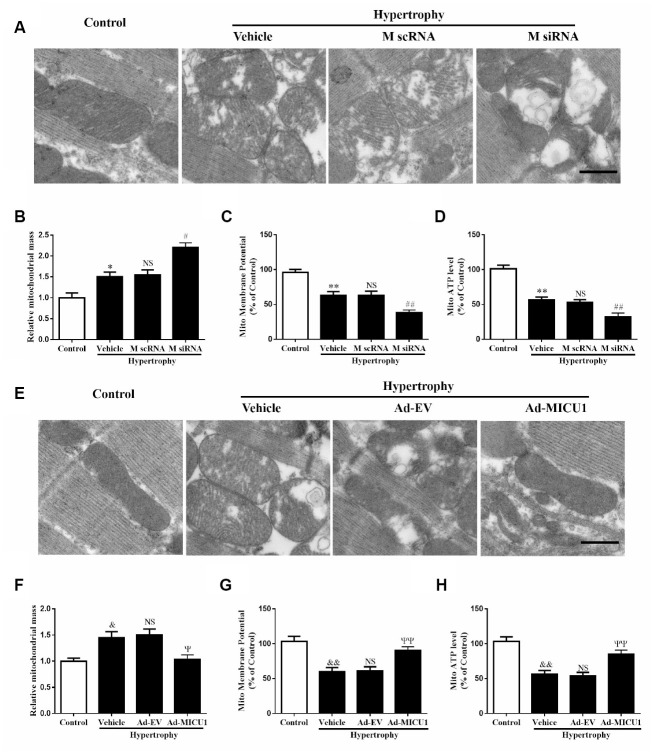
**Mitochondrial disorder contributed to cardiac hypertrophy induced by MICU1 deficiency.** (**A**, **E**) Mitochondrial morphology was observed by transmission electron microscopy in control, MICU1 knockdown (A) and MICU1 overexpression mice (**E**). Scale bars=500 nm. (**B**, **F**) Mitochondrial mass was analyzed by Image J in different mice. (**C**, **G**) Mitochondrial membrane potential (ΔΨm) level was measured by JC-1 kits in different mice. (**D**, **H**) Mitochondrial ATP content was determined by frefly luciferase-based ATP assay kits. All the data represent the means ± SEM. N=6-8/group. ^**^P<0.01 vs. Control (1); ^##^*P*<0.01 vs. M scRNA of Hypertrophy; ^&&^*P*<0.01 vs. Control (2); ^ΨΨ^*P*<0.01 vs. Ad-EV of Hypertrophy.

ROS are primarily generated in mitochondria, and mitochondrial abnormalities induce ROS overproduction. Transmission electron microscopy revealed that MICU1 deficiency was associated with hypertrophy-induced destruction of mitochondrial structure, as evidenced by loss of mitochondrial membranes integrity, unusual vesicle-like structures, completely unstructured cristae, and ambiguous myofilaments (Figure 7A). Quantitative analyses revealed that the knockdown of MICU1 exacerbated the elevation of mitochondrial mass in hypertrophic myocardium (Figure 7B). Furthermore, we found that MICU1 downregulation aggravated the depression of mitochondrial membrane potential (ΔΨm) (Figure 7C), and suppression of ATP content (Figure 7D) in hypertrophic heart. However, MICU1 overexpression rescued hypertrophy-induced mitochondria injury (Figure 7E–7F) and restored the ΔΨm and ATP production (Figure 7G–7H). Therefore, these observations suggested that MICU1 is crucial for the maintenance of mitochondrial morphology and function, and reduced MICU1 expression exacerbated mitochondrial injury in hypertrophic myocardium.

### MICU1 was required for melatonin’s protection against Ang-II-induced cardiac hypertrophy

**Figure 8:**
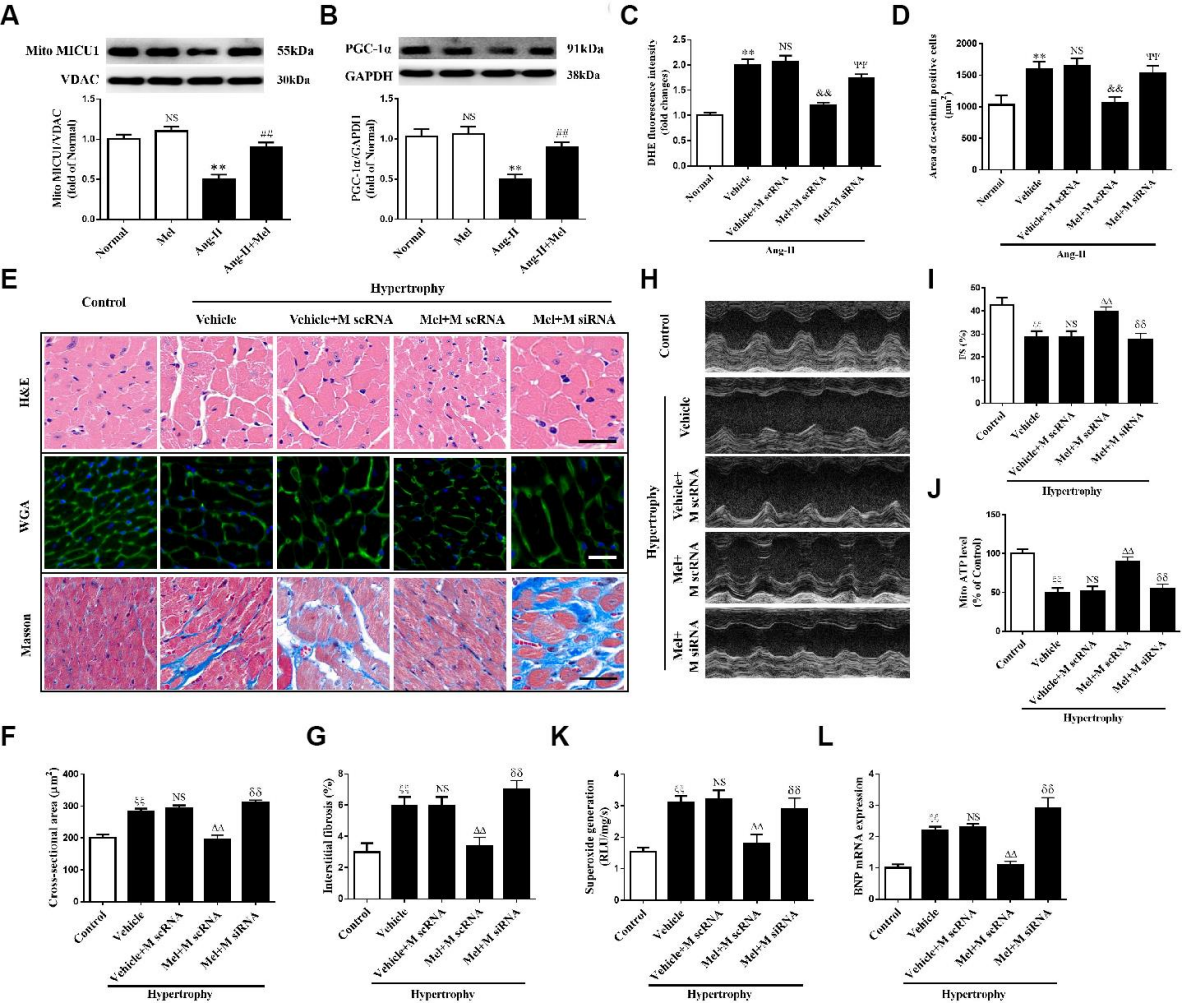
**Melatonin ameliorated Ang-II-induced cardiac hypertrophy by increasing MICU1 pathway.** (**A**, **B**) Western blotting was used to determine expression levels of MICU1 (A) and PGC-1α (B) in NMVMs with the treatment of Ang-II and melatonin. (**C**) ROS generation in NMVMs was measured by DHE staining. (**D**) Cell surface areas were measured in neonatal mice ventricular myocytes (NMVMs). (**E**–**G**) H&E staining and wheat germ agglutinin (WGA) staining were used to measure the enlargement of cardiomyocytes and Masson staining was used to determine interstitial fibrosis. (**H**) Representative echocardiographic image of the left ventricle in different mice was represented. (**I**) FS was used to reflect cardiac function. (**J**) Mitochondrial ATP content was determined by ATP assay kits. (**K**) Superoxide content was quantified with lucigenin-enhanced luminescence. (**L**) The mRNA expression levels of BNP were measured by qRT-PCR. Mel, melatonin. Presented values are means ± SEM. N=6-8/group. ^**^P<0.01 vs. Mel; ^##^*P*<0.01 vs. Ang-II; ^&&^*P*<0.01 vs. (Vehicle + M scRNA) of Ang-II; ^ΨΨ^*P*<0.01 vs. (Mel+ M scRNA) of Ang-II; ^ξξ^*P*<0.01 vs. Control; ^ΔΔ^*P*<0.01 vs. (Vehicle + M scRNA) of Hypertrophy; ^δδ^*P*<0.01 vs. (Mel + M scRNA) of Hypertrophy.

It is acknowledged that melatonin has therapeutic effects in cardiovascular diseases, but whether it can influence MICU1 in Ang-II-induced cardiac hypertrophy is not identified. Melatonin treatment reversed the reduction of MICU1 and PGC-1α in Ang-II-treated NMVMs, but not in NMVMs (Figure 8A, 8B). Melatonin considerably improved ROS, attenuated cardiomyocyte enlargement and decreased the mRNA levels of BNP in hypertrophic NMVMs (Figure 8C, 8D and Supplementary Figure 5A). In conclusion, melatonin may protect against cardiomyocyte hypertrophy via PGC-1α/ MICU1 pathway.

In hypertrophic mice, after 4 weeks of persistent melatonin treatment, melatonin ameliorated the enlargement of cardiomyocytes and interstitial fibrosis, compared with the corresponding controls. Notably, such effects were absent in MICU1 knockdown mice (Figure 8E, 8F). Moreover, chronic treatment of melatonin improved cardiac function recovery in hypertrophic heart, whereas little alterations have taken place in MICU1 downregulation mice (Figure 8H–8I; Supplementary Figure 5B, 5C). Furthermore, melatonin increased mitochondrial ATP level (Figure 8J), reduced superoxide generation (Figure 8K), and attenuated BNP expression (Figure 8L) in the corresponding controls, but not MICU1-deficient mice subjected to cardiac hypertrophy. Hence, MICU1 was indispensable to melatonin-induced protection against cardiac hypertrophy.

## DISCUSSION

In the present study, we have made several observations. First, we demonstrated that MICU1 was downregulated in hypertrophic hearts. Second, our genetic experiments found that MICU1 reduction exacerbated Ang-II-induced cardiac hypertrophy both *in vivo* animal and *in vitro* cellular models; in contrast, the enforced expression of MICU1 inhibited the development of cardiac hypertrophy. Third, we showed that oxidative stress, primarily caused by mitochondrial abnormalities, mediated the adverse effects of MICU1 deficiency in the hypertrophic myocardium. Fourth, melatonin administration increased the expression of PGC-1α/MICU1 and, furthermore, MICU1 was required for the protective effects of melatonin against Ang-II-induced cardiac hypertrophy.

Mitochondria are highly dynamic organelles and their proper function is important for the maintenance of cellular homeostasis [24]. As a protein localized to the inner mitochondrial membrane, MICU1 is the first component of the Ca^2+^ uniporter complex identified through comparative physiology and evolutionary genomic approaches [10]. It has been demonstrated that FOXD1-dependent MICU1 expression regulates mitochondrial activity and cell differentiation [25]. Leng et al. [26] revealed that MICU-related oxidation/antioxidation disequilibrium is strongly involved in intra-abdominal hypertension-induced damage to intestinal barriers. Another study revealed that MICU1 deficiency is associated with mitochondrial Ca^2+^ uptake during aerobic metabolism impairment, muscle weakness and myofiber damage during physical activity [27]. However, the expression/function of MICUs in Ang-II-induced cardiac hypertrophy remains largely unknown yet. In this study, we demonstrated that MICU1 was more remarkably reduced than MICU2 in hypertrophic myocardium. Thus, we hypothesized that MICU1 might serve as the major molecule in the development of myocardial hypertrophy. With genetic method, we found that MICU1 deficiency aggravated myocardial hypertrophy both *in vivo* and *in vitro*. In contrast, upregulation of MICU1 attenuated cardiac hypertrophy. Therefore, our observations provided evidence that downregulated MICU1 in hypertrophic myocardium contributes to Ang-II-induced cardiac hypertrophy. Several studies have found that TGF-β1 mediates the hypertrophic cardiomyocyte growth induced by Ang-II [28–30]. An increasing body of evidence suggests that retinoic acid-related orphan receptor-α (RORα) protects against Ang-II-mediated cardiac hypertrophy [31, 32]. In addition, sirtuins, such as Sirt4 promotes hypertrophic growth, the generation of fibrosis and cardiac dysfunction by increasing ROS levels upon pathological stimulation [33]. Importantly, recent studies demonstrated that MICU1 downregulation in hearts was partly the results of decreased expression of transcriptions factor Sp1 [34]. These findings provide clue for understanding the regulatory mechanisms of MICU1 expression in hypertrophic hearts in our future research.

It is well established that oxidative stress is the common mechanism of pathological cardiac hypertrophy and HF induced by multiple etiologies [35]. The elevated oxidative stress in cardiac hypertrophy and HF can be a consequence of increased ROS generation [36, 37]. Previous study found that Ang-II increase mitochondrial ROS levels in cardiomyocytes, and mitochondrial oxidative stress contributes to Ang-II-mediated cardiac hypertrophy [38]. Therefore, we examined the specific role of oxidative stress in hypertrophic NMVMs. Our results revealed that Ang-II caused a substantial accumulation of ROS/mitoROS in NMVMs, which was aggravated in MICU1 deficiency NMVMs. In contrast, MICU1 upregulation inhibited ROS/mitoROS production. A previous study demonstrated that upregulated MICU1 inhibited cell apoptosis via reducing mitochondrial ROS in diabetic cardiomyocytes [13]. Mitochondria, known as the “the cellular power plants”, are an important source of ROS [39]. In our study, we found that MICU1 reduction significantly aggravated mitochondrial morphological destruction, depressed mitochondrial ATP and ΔΨm in hypertrophic myocardium, whereas enforced MICU1 attenuated these detrimental effects. Taken together, these results supported the conclusion that reduced MICU1 causes abnormal mitochondrial morphology and function, resulting in oxidative stress and pathological cardiac hypertrophy.

Cardiac hypertrophy, the compensatory response of the heart to stress, is characterized by an increase in myocardial mass and protein synthesis, by the excessive deposition of extracellular matrix [40], and by the abnormal expression of fetal genes, such as β-MHC, ANP and BNP accumulating [41, 42]. Angiotensin-converting enzyme inhibitors in combination with calcium antagonists have been verified particularly efficacious in reducing left ventricular hypertrophy [43]. However, these exogenous biosynthetic drugs have unavoidable side effects [44, 45]. Therefore, searching for safer therapies, especially endogenously produced substance, and further illuminating the underlying mechanisms have great significance. Melatonin, the main hormone of the pineal gland, is believed to function in practically every living organism [46]. However, whether melatonin protects against Ang-II-induced cardiac hypertrophy and the potential mechanisms remain elusive. In our study, we found that melatonin attenuated myocardial hypertrophy. It has been reported that melatonin attenuates mitochondrial oxidative damage and prevents mitochondrial dysfunction [47]. Recent studies revealed that MICU1 inhibited ROS-triggered apoptosis by enhancing NAD(P)H produced during the TCA cycle [17]. However, it remains ambiguous whether MICU1 is involved in melatonin’ protection in cardiac hypertrophy. As a well-known transcription factor, PGC-1α plays a pivotal role in the regulation of mitochondrial biogenesis and oxidative metabolism, serves as a prospective target for cardiac hypertrophy [48, 49]. Of note, in the sarcoplasmic reticulum, the overexpression of PGC-1α upregulated the mitochondrial Ca^2+^ uptake uniporter [50]. In our experiments, we found that melatonin increased expression of PGC-1α and MICU1 in the hypertrophic myocardium. Our previous study has demonstrated that melatonin increased the expression of PGC-1α and promoted mitochondrial function in post-myocardial infarction [51]. These observations indicated melatonin may exert protection effects on cardiac hypertrophy by persevering mitochondrial homeostasis. Furthermore, our study proved that melatonin sustained mitochondrial function and reduced ROS production in hypertrophic myocardium, but not in mice with MICU1 knockdown. Conclusively, our study suggested that melatonin could effectively alleviate cardiac hypertrophy through increasing MICU1.

This study has several limitations: first, gene silencing and virus transfection were used in our study without the application of transgenic mouse; second, not all the conclusions were derived from *in vivo* study. Despite these limitations, we believe that our study provides important new insights into pathological cardiac hypertrophy.

**Figure 9:**
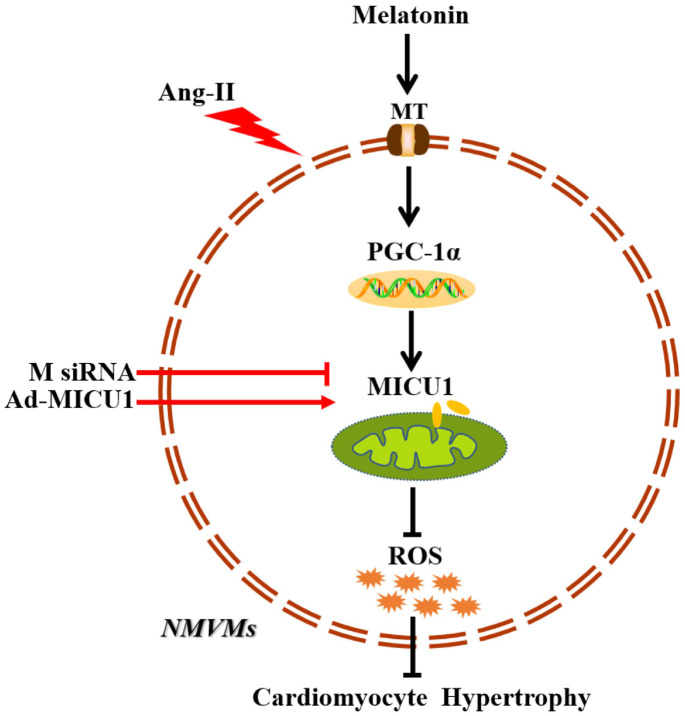
**Schematic diagram depicts that melatonin ameliorates cardiac hypertrophy by activating MICU1.** As located in the intermembrane space of mitochondria, MICU1 is responsible for maintaining mitochondrial homeostasis. Exposure to Ang-II, MICU1 is significantly downregulated in NMVMs. With genetic methods, we found that MICU1 reduction in cardiomyocytes leads to mitochondrial dysfunction, enhances ROS overload and subsequently aggravated cardiomyocyte hypertrophy, but not in MICU1 overexpression NMVMs. As a therapeutic agent, melatonin is able to increase MICU1 expression via activating PGC-1α to maintain mitochondrial homeostasis and attenuate ROS overload, consequently ameliorated cardiomyocyte hypertrophy. MT, melatonin receptor; MICU1, mitochondrial calcium uptake 1; PGC-1α, peroxisome proliferator-activated receptor-γ coactivator-1α; ROS, reactive oxygen species; NMVMs, neonatal mice ventricular myocytes; M siRNA, MICU1-specific siRNA; Ad-MICU1, recombinant adenovirus encoding MICU1.

In summary, the present study provides evidence that downregulation of MICU1 aggravates cardiac hypertrophy, and enforced MICU1 attenuates myocardial hypertrophy via ameliorating mitochondrial injury and inhibiting ROS generation. Melatonin exerts a protection effect on cardiac hypertrophy through PGC-1α/MICU1 pathway (Figure 9). These findings will inspire people to design clinical trials to restore balance of body melatonin secretion and to develop new intervention means targeting at MICU1 in HF patients.

## MATERIALS AND METHODS

### Animals

The C57BL6/J mice (male, 8-10 weeks old) were purchased (Vital River, Beijing, China) for our study. Mice were housed in a room under controlled temperature (22-24° C) and were fed standard rodent chow with free access to water. All experiments performed in adherence to the National Institutes of Health Guidelines on the Use of Laboratory Animals (Bethesda, MD, USA) and conformed to the Institutional Animal Care and Use Committee of The General Hospital of Western Theater Command.

### Angiotensin-II administration

Animals were anesthetized with 2% isoflurane and mini-osmotic pumps (ALZET, model 1004, Durect, Cupertino, CA, USA) releasing either Ang-II (1000 ng/kg/min) (Sigma-Aldrich, St. Louis, MO, USA) or saline were inserted underneath the mice skin via mid-scapular incision subcutaneously. After 28 days of Ang-II infusion, mice were killed with excessive CO_2_ inhalation.

### Experimental protocols

Mice were randomly divided into groups of Control and Hypertrophy. Animals in Hypertrophy group were all treated with Ang-II infusion and were further divided into groups as follows: (1) Hypertrophy+Vehicle (2% ethanol, ip), (2) Hypertrophy+M scRNA (scrambled siRNA via intramyocardial injection), (3) Hypertrophy+M siRNA (MICU1 siRNA via intramyocardial injection), (4) Hypertrophy+Ad-EV (control adenovirus via intramyocardial injection), (5) Hypertrophy+Ad-MICU1 (recombinant adenovirus expressing MICU1 via intramyocardial injection), (6) Hypertrophy+Mel (melatonin,10 mg/kg/day, ip, 4 weeks before Hypertrophy), (7) Hypertrophy+Vehicle+M scRNA (scrambled siRNA and vehicle), (8) Hypertrophy+Mel+M scRNA (scrambled scRNA and melatonin) and (9) Hypertrophy+Mel+M siRNA (MICU1 siRNA and melatonin). NMVMs were randomly assigned to Normal, Normal+Mel, Ang-II, Ang+Mel, Vehicle (PBS), M scRNA (scrambled siRNA), M siRNA (MICU1 siRNA), Ad-EV (control adenovirus) and Ad-MICU1 (recombinant adenovirus expressing MICU1).

### Knockdown expression of MICU1

siRNA against MICU1 and scrambled siRNA were designed and synthesized by GenePharma Company (Shanghai, China). The sequences of siRNAs are provided in Supplementary Table 2. Mice were anesthetized with 2% isoflurane to expose hearts under aseptic conditions. siRNAs (20 μg diluted in 30 μl vivo-jetPEITM and 10% glucose mixture) were delivered via intramyocardial injection into the apex and anterolateral wall with a 30-gauge needle. *In vitro*, all transfections were carried out using Lipofectamine RNAiMAX (Thermo Fisher Scientific, Waltham, MA, USA). NMVMs were cultured in medium containing 20 μg of MICU1 siRNA or scrambled siRNA for 6 h per day on two consecutive days. After 2 days injection or incubation, the transfection efficiency was determined by Western blotting [12].

### Forced expression of MICU1

Recombinant adenovirus overexpressing mouse MICU1 (NM_001291442.1) were also designed and purchased from GenePharma Company (Shanghai, China). The Adeno-X Rapid Titer Kit (Clontech Laboratories, Mountain View, CA, USA) were used to detect the viral titer. A total of 30 μl adenovirus (1.5× 10^10^ ifu/ml) were delivered via intramyocardial injection into the apex and anterolateral wall (10 μl at each of three sites) with a 30-gauge needle [13]. NMVMs were plated onto slides in 24-well plates and allowed to reach 50-70% confluence at the time of transfection. Then, NMVMs were incubated in growth medium with adenoviruses (multiplicity of infection=50) for 2 h at 37° C, and then were grown in new medium for another 48 h at 37° C. After 2 days injection or incubation, the overexpression efficiency was also detected by Western blotting. The coding sequences of MICU1 were amplified using the primers shown in Supplementary Table 3.

### Measurement of blood pressure

The systolic and diastolic blood pressures were obtained as described previously [52]. Briefly, after intervention, the mice were restrained by a noninvasive tail-cuff plethysmography (BP-2010A; Sofron Biotechnology, Beijing, China). Mice were placed in plastic restrainers. A cuff with a pneumatic pulse sensor was attached to the tail. Mice were allowed to habituate to this procedure for 7 days before the actual measurement. The blood pressure measurement experiments were conducted in a designated quiet area (22 ± 2° C), where mice acclimatized for a 1-hour period before experiments began.

### Cardiac function

After 28 days of Ang-II infusion, mice were anesthetized with inhalation of isoflurane at a concentration of 2% and placed on a heating pad to maintain their body temperature. For echocardiography measurements, M-mode tracings derived from the short axis at the papillary muscle level and the parasternal long axis of the left ventricular were recorded using a Mylab30CV ultrasound system (Biosound Esaote Inc.) equipped with 15-MHz probe. Then, LVEDd and LVESd were measured at the time of the largest and smallest left ventricular areas, respectively. FS was calculated using the following formula: FS (%) = (LVEDd-LVESd)/LVEDd×100% [42].

### Histological analysis

After the mice were euthanized, their HW, TL, and LVW were determined. From these data, the ratios of HW/TL and LVW/TL were calculated. Part of each heart tissue was fixed in 4% paraformaldehyde and was subsequently dehydrated in 70% alcohol, followed by embedding in paraffin wax. Sections (5 μm thickness) were prepared and stained with H&E or WGA (green; Sigma-Aldrich) for the evaluation of myocyte size. Masson staining (St. Louis, MO, USA) was used to measure the cardiac interstitial fibrosis.

### Analysis of mitochondrial morphology

According to the manufacturer’s instructions, mitochondria were isolated from cardiomyocytes or hearts using the Mitochondria Isolation Kit (Beyotime, Shanghai, China). Cardiac tissues were fixed in 4% paraformaldehyde overnight at 4° C, and were post-fixed in 1% osmium tetroxide for 1 h, conformed to standard protocols. The sections were observed by JEM-1230 transmission electron microscope (Hitachi H-600IV, Hitachi, Tokyo, Japan). Mitochondrial mass was analyzed using Image J [53].

### Measurement of ATP content

ATP content was measured by the Apoglow luciferin-luciferase bioluminescence kit (Cambrex, East Rutherford, NJ, USA) according to the manufacturer’s instructions. Cardiac tissue samples were homogenized and centrifuged. Supernatants were mixed with the ATP detection working solution in a white 96-well plate. Standard curves were also generated, and the protein concentration in each treatment group was determined using the Bradford protein assay. Total ATP levels were expressed as nmol/mg protein.

### Assessment of ΔΨm

ΔΨm in myocardium was assessed using JC-1 kit (Invitrogen, Carlsbad, CA, USA). Cardiomyocytes isolated from mice were seeded on gelatin-coated culture chamber slides and stained with JC-1 (5 μM) at 37° C for 10 min. Then, Cells were rinsed with the HEPES-saline buffer. The results in fluorescence intensity were expressed as the ratio of 590 to 530 nm emission using a spectrofluorimeter (Spectra Max Atlanta, GA, USA).

### Quantification of superoxide production

Myocardial superoxide content was measured by lucigenin-enhanced luminescence. Cardiac tissues were weighed, cut into uniform cubes (0.5 mm^3^), and then transferred into a polypropylene tube containing 1mL PBS and lucigenin (Sigma, 0.25 mmol/L). The tube was placed in a FB12-Berthold luminometer (Berthold Technologies, Bad Wildbad, Germany). The RLU emitted was recorded and integrated over 30 seconds intervals for 5 min. Activity was normalized with dry tissue weights [54].

### Cell culture

Primary cardiomyocytes (NMVMs) were prepared from the hearts of newborn C57BL6/J mice (1-2 days old), as previously described [55]. The ventricular heart was quickly removed, cut into small chunks and washed with Hanks’s balanced salt solution (HBSS) without Ca^2+^ and Mg^2+^ (Gibco, Invitrogen, Carlsbad, CA, USA). Then, the tissue was minced in trypsin-EDTA 0.125% (Invitrogen, Carlsbad, CA, USA) at 4° C overnights. Subsequently, tissues were digested with collagenase (Invitrogen, 0.5 mg/ml in Dulbecco’s modified Eagle’s medium, DMEM) in a shaking bath at 37° C. After 1.5 h, NMVMs were collected and cultured in DMEM with 25 mM glucose, 10% heat-inactivated horse serum, 10% heat-inactivated fetal bovine serum, and 1000 units/ml penicillin/streptomycin. NMVMs were treated with a solvent carrier (control) or 200 nM Ang-II (Solarbio Biotechnology) for 48 h in the presence or absence of selected pharmacological reagents. NMVMs were transfected with MICU1 siRNA, scrambled siRNA, Ad-MICU1, or Ad-EV. Experiments were performed with these cells at 48 h post-transfection.

### Measurement of cell surface area

The cells were fixed by 4% formaldehyde followed by 5 min of permeabilization with 0.2% Triton X-100. Then, cells were stained with α-actinin (Invitrogen, Carlsbad, CA, USA), followed by staining with a fluorescent secondary antibody. Immunofluorescence images were acquired by Olympus laser confocal microscope (FV 1000, Olympus, Tokyo, Japan). Cardiomyocytes surface area was determined from more than 100 cells each group using Image-Pro Plus software (Media Cybernetics, Rockville, MD, USA) by total cell area divided total cell number.

### Detection of ROS generation

DHE staining was used to detect the *in situ* formation of superoxide according to the oxidative fluorescent microtopography, as described previously [56]. DHE staining was visualized under a laser scanning confocal microscope (FV 1000, Olympus, Tokyo, Japan) and then images of NMVMs were analyzed with Image-Pro Plus software version 6.0 (Media Cybernetics, Rockville, MD, USA). The mean fluorescence intensity of each cell was calculated, and the total cell emission signals per field were averaged for data analysis. ROS was also determined by using the ROS ELISA kit (Elabscience). MitoROS also were detected using the fluorescent probe MitoSOX (Invitrogen), according to the manufacture’s protocols. Images were captured by a laser confocal microscope (FV 1000, Olympus, Tokyo, Japan) and Image-Pro image analysis software.

### Determination of mRNA expression

RNAs from myocardium or NMVMs were extracted by TRIzol (Invitrogen, Carlsbad, CA, USA). A total 2 μg of RNA was reverse-transcribed into complementary DNA. The level of mRNAs was quantified by quantitative real-time polymerase chain reaction (qRT-PCR) using SYBRGreen Master Mix (Takara, Dalian, Japan). The target gene expression was normalized to 18s ribosomal RNA, which served as an internal control for total complementary DNA content. Primer sequences are shown in Supplementary Table 4.

### Measurement of protein expression

Cardiac tissues were lysed in RIPA buffer. Protein concentration was quantified by bicinchoninic acid (BCA) assay (Bio-Rad, Hercules, CA, USA). Equal amounts of protein (approximately 20 μg) were separated by 10% SDS-PAGE, and then transferred to Polyvinylidene Fluoride (PVDF) membranes (Bio-Rad, Hercules, CA, USA). The membranes were incubated with primary antibodies overnight at 4° C. More details of antibodies are shown in Supplementary Table 5. The transblotted membranes were washed and incubated with secondary antibodies for 1 h at room temperature. The amounts of proteins were verified by immunoblotting for GAPDH (1:5000) and VDAC (1: 5000). The blots were visualized using chemiluminescence and quantified using Image-Pro Plus 6.0 (Media Cybernetics) [51]. One of the most accepted and trusted methods for antibody specificity is knockout verification [57].

### Statistical analysis

The data are expressed as means ± SEM. The data of Western blotting were analyzed by Kruskal-Wallis test and Dunn post-hoc test. Other comparisons were made by one-way ANOVA, and the post-hoc test was performed by Bonferroni correction. *P*<0.05 was considered to be statistically significant. All statistical analysis was performed by GraphPad Prism software version 6.0 (GraphPad Software, La Jolla, CA, USA).

## Supplementary Material

Supplementary Figures

Supplementary Tables
