# Supplementary material for: Melatonin alleviates angiotensin-II-induced cardiac hypertrophy via activating MICU1 pathway
Source: Aging (Albany NY). 2020 Nov 26;13(1):493–515. doi: 10.18632/aging.202159 (PMC7834983; doi:10.18632/aging.202159)
Supplement: Supplementary Figures [file aging-13-202159-s001.pdf]

SUPPLEMENTARY FIGURES

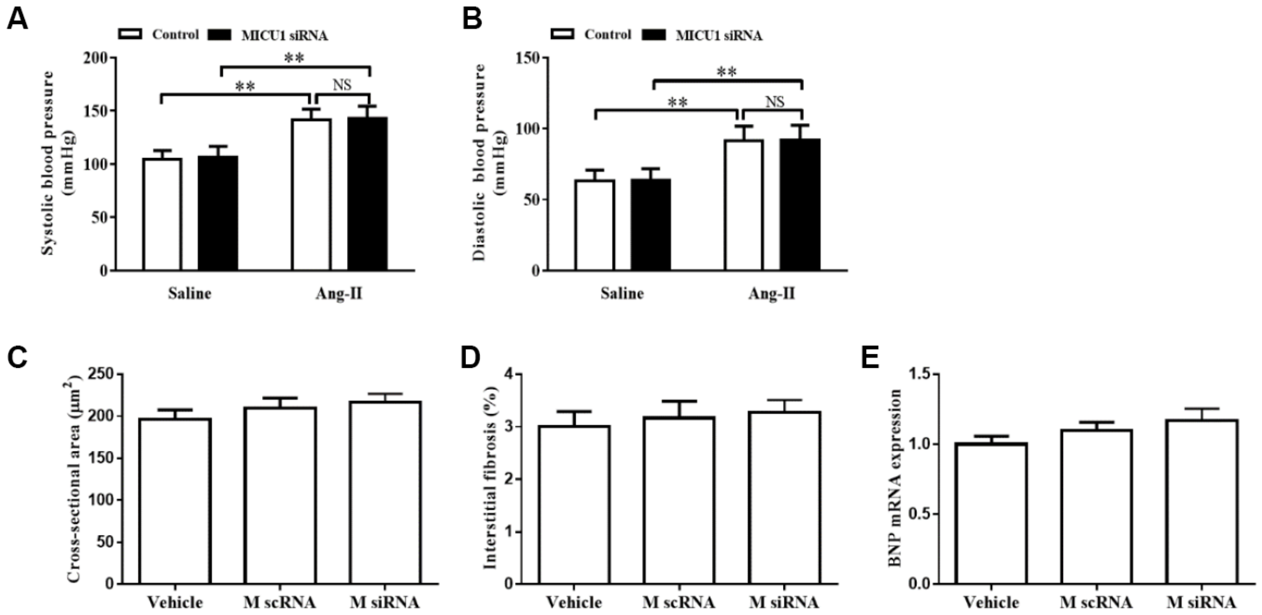

Supplementary Figure 1. The blood pressure and hypertrophic related indicators in MICU1 knockdown mice was determined. (**A**, **B**) The systolic blood pressure (SBP) and diastolic blood pressure (DBP) were determined by a noninvasive tail-cuff plethysmography. (**C**) Cross-sectional cardiomyocyte areas were summarized. (**D**) The interstitial fibrosis was quantified. (**E**) The mRNA expression of BNP was detected by qRT-PCR.M scRNA, scrambled siRNA; M siRNA, MICU1-specific siRNA. Presented values are means ± SEM. N=6-8/group. **P<0.01 vs. Control in Saline.

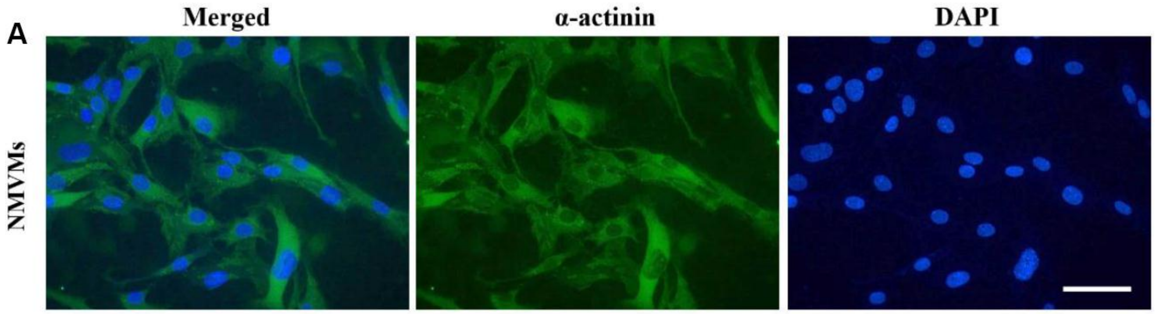

Supplementary Figure 2. Positive immunofluorescence staining of NMVMs. (**A**) The isolated neonatal mice ventricular myocytes NMVMs were authenticated by α-actinin staining. Scale bars=10 μm.

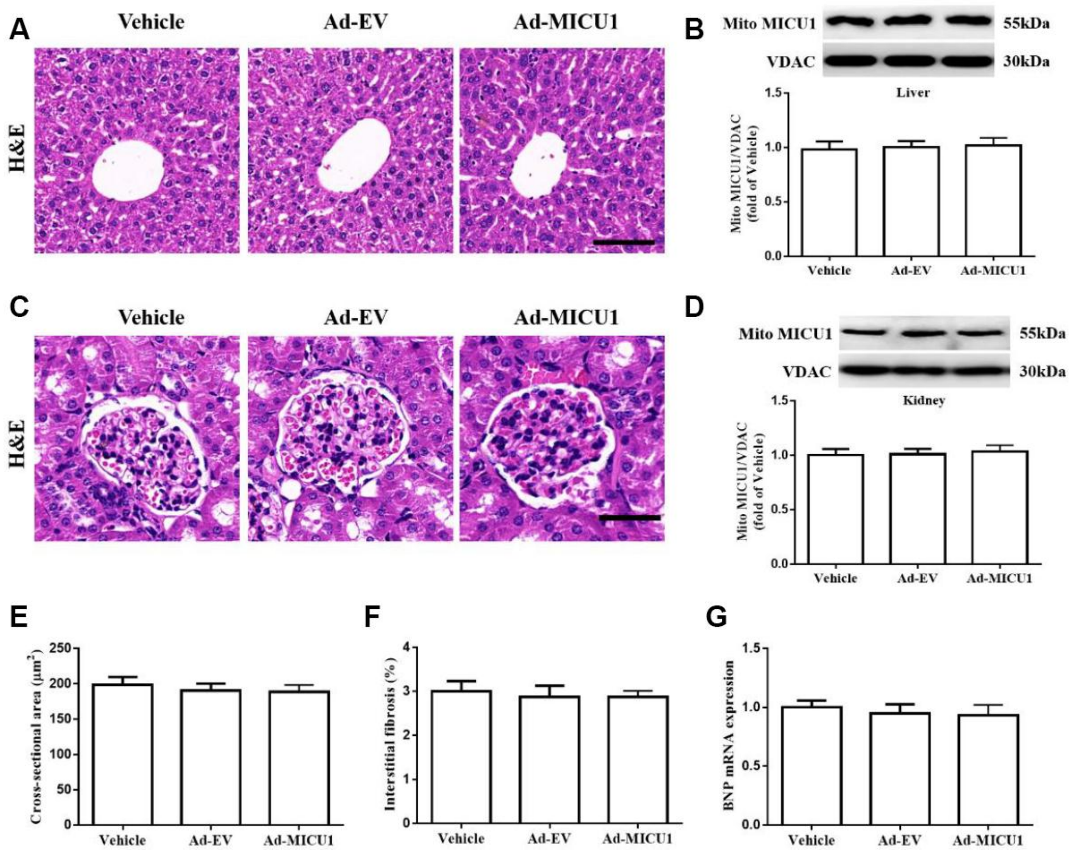

Supplementary Figure 3. The effects and efficiency of MICU1 adenovirus in control mice. (**A**, **C**) The structure of liver (**A**) and kidney (**C**) was observed by H&E staining after intramyocardial injection of adenovirus in control mice. (**B**, **D**) The protein levels of MICU1 in liver (**B**) and kidney (**D**) after intramyocardial injection of adenovirus in control mice was detected by western blotting. (**E**) Cross-sectional cardiomyocyte areas were summarized. (**F**) The interstitial fibrosis was quantified. (**G**) The mRNA expression of BNP was measured by qRT-PCR. Ad-EV, control adenovirus; Ad-MICU1, recombinant adenovirus encoding MICU1. Presented values are means ± SEM. N=6-8/group.

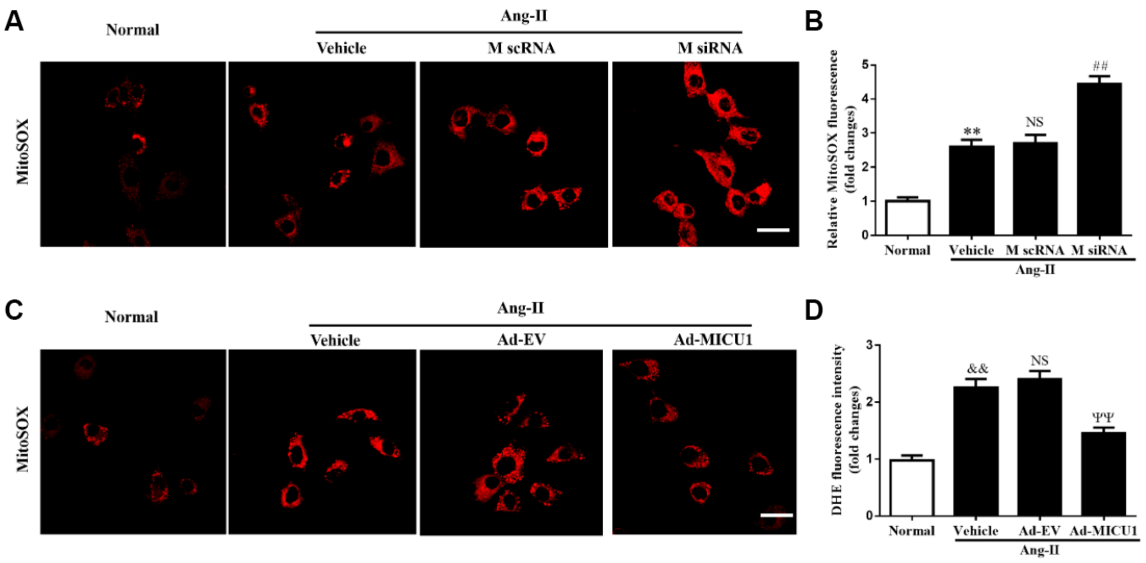

Supplementary Figure 4. MICU1 mediated cardiomyocyte hypertrophy by modulating oxidation states. (**A**, **B**) The mitochondrial ROS levels in cardiomyocytes treated with Ang-II and MICU1 siRNA were analyzed by fluorescent probe MitoSOX. Representative confocal microscope images (**A**) and fluorescence quantitation (**B**) were presented. Scale bars=20 μm. (**C**, **D**) The mitochondrial ROS levels in NMVMs treated with Ang-II and Ad-MICU1 were analyzed by fluorescent probe MitoSOX. Representative confocal microscope images (**D**) and fluorescence quantitation were presented. Scale bars=20 μm. Presented values are means ± SEM. N=6-8/group. $^{**}P$<0.01 vs. Normal (1); $^{\#\#}P$<0.01 vs. M scRNA of Ang-II; $^{\&\&}P$<0.01 vs. Normal (2); $^{\Psi\Psi}P$<0.01 vs. Ad-EV of Ang-II.

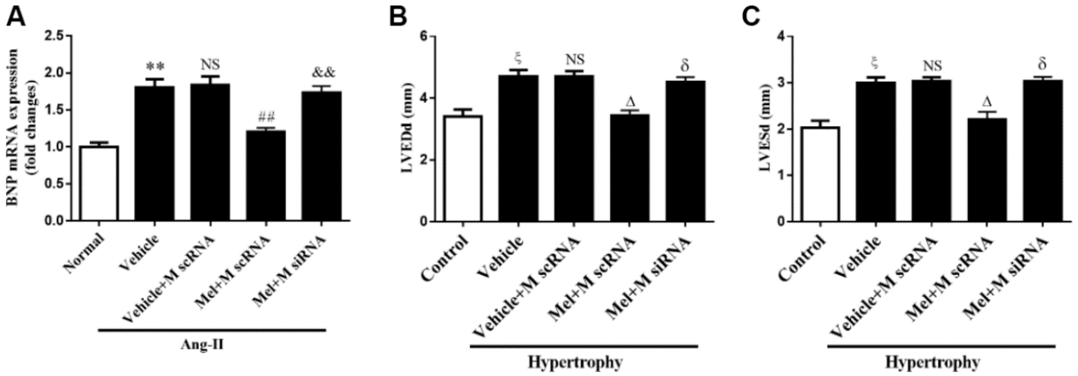

Supplementary Figure 5. The effects of melatonin on BNP, LVEDd and LVESd. (**A**) The mRNA expression of BNP was measured by qRT-PCR. (**B**, **C**) Echocardiographic assessment of left ventricular end-diastolic dimension (LVEDd) and left ventricular end-systolic dimension (LVESd) was used to reflect cardiac function. Data represent the means ± SEM. N=6-8/group. $^{**}P$<0.01 vs. Normal; $^{\#\#}P$<0.01 vs. (Vehicle + M scRNA) of Ang-II; $^{\&\&}P$<0.01 vs. (Mel + M scRNA) of Ang-II; $^{\xi}$*P<0.05 vs. Control;* $^{\Delta}P$<0.05 vs. (Vehicle + M scRNA) of Hypertrophy; $^{\delta}P$<0.05 vs. (Mel + M scRNA) of Hypertrophy.
