# Supplementary material for: Melatonin alleviates angiotensin-II-induced cardiac hypertrophy via activating MICU1 pathway
Source: Aging (Albany NY). 2020 Nov 26;13(1):493–515. doi: 10.18632/aging.202159 (PMC7834983; doi:10.18632/aging.202159)
Supplement: Supplementary Tables [file aging-13-202159-s002.pdf]

SUPPLEMENTARY TABLES

Supplementary Table 1. Basal physiological parameters in all kinds of mice.

Parameter	Control	MICU1 knockdown	MICU1 overexpression
HR (bpm)	410±26	417±23	400±25
HW/TL (mg/cm)	60±8.0	62±9.0	61±8.5
LVW/TL (mg/cm)	50±7.0	52±8.0	51±6.0
LVEDd (mm)	3.4±0.4	3.6±0.3	3.5±0.2
LVESd (mm)	2.0±0.2	2.3±0.1	2.2±0.2
FS (%)	45±2.5	47±3.0	46±1.5

Note: MICU1, mitochondrial calcium uptake 1; HR, heart rate; HW/TL, heart weight to tibia length; LVW/TL, left ventricular weight to TL; LVEDd, left ventricular end-diastolic dimension; LVESd, left ventricular end-systolic dimension; FS, fractional shortening.

Supplementary Table 2. Sequences of siRNA.

siRNA	Forward (5'-3')	Reverse (5'-3')
MICU1	GCCACCUUGAAAGUAAUCATT	UGAUUACUUUCAAGGUGGCTT
Control	UUCUCCGAACGUGUCACGUTT	ACGUGACACGUUCGGAGAATT

Supplementary Table 3. Sequences of primers used in gene cloning.

Gene symbol		Primers sequences	Product size (bp)
MICU1	forward primer	CTGGAAGATCTCGGCATTCTCCGAG	1914
	reverse primer	GACGCGTCGACCTGCCAGCTCATTTC	

Supplementary Table 4. Primer sequences used for PCR.

Gene	Forward (5'-3')	Reserve (5'-3')
ANP	GTGTACAGTGCGGTGTCCAA	ACCTCATCTTCTACCGGCAT
BNP	GAGGTCACTCCTATCCTCTGG	GCCATTTCCTCCGACTTTTCT
β-MHC	CCGAGTCCCAGGTCAACAA	CTTCACGGGCACCCTTGGA
18sRNA	CGCGGTTCTATTTTGTTGGTTT	GCGCCGGTCCAAGAATTT

Supplementary Table 5. Primary antibodies used for Western blot and immunofluorescence.

Antibody	Company (Cat. No.)	Working dilutions
MICU1	Abcam (ab190114)	WB: 1:500
MICU2	Abcam (ab101465)	WB: 1:500
ANP	ThermoFisher Scientific (PA5-72527)	WB: 1:200
BNP	ThermoFisher Scientific (PA5-79760)	WB: 1:200
β-MHC	ThermoFisher Scientific (PA5-76225)	WB: 1:200
PGC-1α	Abcam (ab54481)	WB: 1:500
VDAC	Abcam (ab14734)	WB: 1:5000
GAPDH	Abcam (ab181602)	WB: 1:5000
α-actinin	Abcam (ab32575)	IF: 1:500
